# Paradoxical effects of arsenic in the lungs

**DOI:** 10.1186/s12199-021-00998-2

**Published:** 2021-08-13

**Authors:** Caixia Ren, Yang Zhou, Wenwen Liu, Qi Wang

**Affiliations:** 1 Department of Respiratory Medicine, The Second Hospital of Dalian Medical University, Dalian, 116023 China; 2 Liaoning Clinical Research Center for Lung Cancer, The Second Hospital of Dalian Medical University, Dalian, 116023 China

**Keywords:** Arsenic, Lung, Pathogenic effect, Therapeutic effect

## Abstract

High levels (> 100 ug/L) of arsenic are known to cause lung cancer; however, whether low (≤ 10 ug/L) and medium (10 to 100 ug/L) doses of arsenic will cause lung cancer or other lung diseases, and whether arsenic has dose-dependent or threshold effects, remains unknown. Summarizing the results of previous studies, we infer that low- and medium-concentration arsenic cause lung diseases in a dose-dependent manner. Arsenic trioxide (ATO) is recognized as a chemotherapeutic drug for acute promyelocytic leukemia (APL), also having a significant effect on lung cancer. The anti-lung cancer mechanisms of ATO include inhibition of proliferation, promotion of apoptosis, anti-angiogenesis, and inhibition of tumor metastasis. In this review, we summarized the role of arsenic in lung disease from both pathogenic and therapeutic perspectives. Understanding the paradoxical effects of arsenic in the lungs may provide some ideas for further research on the occurrence and treatment of lung diseases.

## Introduction

Arsenic, an important environmental contaminant, exists all over the world in the form of inorganic arsenic compounds. The most common forms are arsenite and arsenate [1]. Arsenic influences people via water, air, and food. Water is the main source of arsenic exposure [2]. Despite World Health Organization (WHO) stipulations relating to arsenic concentration in water (< 10 μg/L), according to surveys, there are still over 200 million people worldwide exposed to excessive arsenic-contaminated groundwater [3].

The lung is one of the main target organs. A high concentration of arsenic exposure is closely related to lung cancer, as has been confirmed by epidemiological statistical analysis in China [4], the USA [5], Italy [6], and Chile [7]. The mortality rate among those exposed to high doses (> 100 μg/L) of arsenic was higher than unexposed people after stopping exposure for 30–40 years [8, 9]. However, whether low (≤ 10 μg/L) to medium concentrations of arsenic exposure can cause cancer remains controversial, and whether its toxicity is dose dependent is still unknown. Most studies reported that low to medium (10–100 μg/L) concentrations of arsenic exposure is related to cancers [10]. Low doses, even those less than 10 μg/L, can increase the risk of cancer and mortality from chronic disease [6]; thus, The Netherlands is committed to reducing the concentration in tap water to 1 μg/L [11], but there remain many others of different opinions: their studies show that arsenic concentrations below 100–150 μg/L will not increase the risk of lung cancer [12]. There is still no conclusion as to whether low to medium concentrations of arsenic are carcinogenic due to limitations of the epidemiological research to date. In addition, there are many other studies that show arsenic exposure is closely related to lung diseases besides lung cancer, such as pneumonia [13], bronchiectasis [14], asthma [15], and so on.

Interestingly, arsenic, especially As_2_O_3_, plays an important role in the treatment of a variety of tumors, including lung cancer [16], intestinal cancer [17], and especially in leukemia [18], with therapeutic effects that have been widely recognized [19], and several review papers on the therapeutic role of As_2_O_3_ in tumors have been published, covering subjects such as curing APL [20], treating myelodysplastic syndromes, and relapsed/refractory multiple myeloma [21, 22]. As_2_O_3_ plays a therapeutic role not only in hematologic diseases but also in solid tumors [23], although there is no comprehensive summary of As_2_O_3_ and its efficacy in the treatment of lung diseases.

Thus, what role does arsenic play in lung diseases? This review summarized the pathogenic and therapeutic effects in the treatment of lung diseases induced by arsenic under low to medium concentration doses. A discussion of the paradoxical effect of arsenic at the same time may have great significance for the occurrence and treatment of lung diseases.

## Pathogenic effects

As is known, a high concentration of arsenic is toxic and pathogenic, especially in the lungs. Most research reports show that the incidence and mortality of lung cancer in high-arsenic exposure groups are significantly higher than those in the general population or the low arsenic exposure group [24–26]; however, whether low-level arsenic exposure is pathogenic, and whether there is a threshold for arsenic pathogenicity or a dose-response relationship remain to be explored. Besides, inorganic forms of arsenic can be converted into monomethylarsonic acid (MMA) and dimethylarsinic acid (DMA) through methylation-type metabolism in the human body; different forms of arsenic may affect the occurrence of lung diseases in different ways. Therefore, the study of arsenic metabolism may help to understand the role of arsenic in pathogenicity and may provide clues as to the pathogenic mechanism of action of arsenic.

### Epidemiology of arsenic and lung cancer

Arsenic can increase the risk of lung cancer, especially at high concentrations. The relationship between arsenic and lung disease was first found because of the widespread use of arsenic in industries [27]. Subsequently, more epidemiological evidence relates exposure to arsenic to lung cancer [28–30]. Minerals and tobacco, even water and foodstuffs, contain appreciable amounts of arsenic [31], making it necessary to study the relationship between arsenic and lung cancer from an epidemiological perspective. At high concentrations, the higher the exposure concentration, the greater the risk of lung cancer [25, 32, 33]. After the WHO set the maximum arsenic concentration in water at 10 μg/L, various regions have adjusted their allowable concentration of arsenic in water. The risk of lung cancer in high arsenic concentration decreased after intervention [34–37]; therefore, we believe that high arsenic exposure is related to the occurrence of lung cancer. As for medium- and low-concentration arsenic exposure, the results seem to differ. Do medium and low concentrations of arsenic affect the incidence of lung cancer? We sought and analyzed studies on the relationship between low to medium concentrations of arsenic and lung cancer (Table 1).

**Table 1:** Studies of the low to medium arsenic water concentration exposure and lung cancer risk

Country/region	Arsenic concentration (μg/L)	Evaluation index	Outcome	Conclusion
North-west China [38]	7.61–9.25	Lifetime lung cancer risks	3.54 × 10^−5^	Related
Central Italy [6]	19.3 (mean)	HR	Lung cancer (HR = 2.6 male; HR = 2.09 female)	Related
The USA [37]	6.91–13.32 > 13.32	RR	0.94 (0.51–1.72) 1.82 (1.00–3.31)	Related
Northern Chile [39]	< 11, 11–90, 91-335	OR (95% CI)	1.00, 1.27 (0.81–1.98), 2.00 (1.24–3.24)	Related
Northern Chile [7]	10-29, 30–49, 50–199	OR (95% CI)	1.60 (0.50–5.30), 3.90 (1.20–12.30), 5.20 (2.30–11.70)	Related
The USA [12]	3-59	SMR	0.9	Unrelated
The USA [40]	10, 11–84, > 85	RR (95% CI)	1.00,0.75 (0.45–1.25), 0.84 (0.41–1.72)	Unrelated
North-eastern Taiwan [41]	10–49.9, 50–99.9	RR (95% CI)	1.10 (0.74–1.63) 0.99 (0.59–1.68)	Unrelated
Bangladesh [42]	0–10, 11–50, 51–100	RR (95% CI)	1.00, 0.90 (0.62–1.33), 1.10 (0.62–1.96)	Unrelated

*SMR*, standardized mortality ratio; *OR*, odds ratio; *CI*, confidence interval; *RR*, relative risk; *HR*, hazard ratio

As shown previously, the results vary: five studies show that arsenic exposure at low to medium concentrations is related to the occurrence of lung cancer, while the other four consider it unrelated. Through the analysis of the results, the lifetime lung cancer risks were assessed in different age groups, with arsenic exposure at concentrations from 7.61 to 9.25 μg/L. The result showed that the lifetime risk of lung cancer was 3.54 × 10^−5^ and the lung cancer burden was 1.20 × 10^−5^ per person-year (ppy) [38]. Under the average arsenic concentration of 19.3 μg/L, the mortality risk of lung cancer was found to be 2.61 in men and 2.09 in women [6]. Though there are differences between the sexes, the risk ratios differ significantly compared with the non-exposed group, that is, low to medium-concentration arsenic exposure is related to the occurrence and mortality increase of lung cancer. In addition, there are several studies devoted to exploring the dose-dependent nature of the relationship between arsenic exposure and lung cancer. With the increase of arsenic exposure concentration, the incidence and mortality of lung cancer are also increasing. A case-control study found that lung cancer odds ratios (ORs) and 95% confidence intervals (CIs) for the groups exposed to 10–29 μg/L, 30–49 μg/L, and 50–199 μg/L arsenic were 1.60 (0.50–5.30), 3.90 (1.20–12.30), and 5.20 (2.30–11.70) respectively [7]. Similarly, studies in the USA and northern Chile have also found that the incidence of lung cancer increases with increasing arsenic exposure levels [37, 39]. Besides, we found that the occurrence of lung cancer is different across genders. Some studies have shown that the risk of lung cancer and the burden of disease in men are higher than in women [6, 43], indicating that there may be gender differences in the carcinogenic risk of arsenic exposure. In addition to the five epidemiological studies that found the exposure to low to medium concentrations of arsenic is related to lung cancer, we analyzed the other four studies that are unrelated. A study in north-eastern Taiwan found that the relative risk (RR) of lung cancer was 1.10 (95% CI: 0.74–1.63) and 0.99 (95% CI: 0.59–1.68) after arsenic exposure concentration of 10–49.9 μg/L and 50–99.9 μg/L, respectively, suggesting that there is no significant correlation between low to medium concentration arsenic exposure and lung cancer [41]. In this study, a total of 178 newly diagnosed lung cancer cases were identified. After adjusting for variables including age, gender, years of schooling, cigarette smoking status, and habitual alcohol consumption, different histological types show different RR. In squamous cell carcinomas, the RR was 0.53 and 1.32, whereas in other types, the RR in the 50–99.9 μg/L group was lower than that in the 10–49.9 μg/L group, so those lung cancers caused by arsenic may be related to specific tumor histological types. Besides, the residents involved in this study were followed for 11 years, and although lacking in statistical precision, the risk of lung cancer increased with the exposure time, which means that the exposure period is also a vital factor requiring evaluation. While in another study, the standardized mortality ratio (SMR) was 0.9 in some parts of the USA, where the concentration of arsenic in drinking water was between 3–59 μg/L. Studying this article, we found that the RR was 1.00, 1.0, and 0.98/0.97 (males and females) with a median arsenic exposure concentration of 3.0 μg/L, 3.1–9.9 μg/L, and 10–59.9 μg/L, respectively, that is, the arsenic in underground water is unrelated to mortality as a result of lung cancer [12]. We did also find that in this study, the authors only adjusted for age and ethnicity. Cigarette smoking and occupational exposure also played a significant role in the occurrence of lung cancer. Considering that RR is around 1.0, we believe that further adjustments to factors related to lung cancer such as cigarette smoking may obtain more accurate results. Similarly, in the other two studies, they both think that arsenic in low to medium concentrations is unrelated to lung cancer, but we found that the lack of data of early exposure in a mobile population, the insufficient number of samples, and short exposure time may have a significant influence on the results. In overview, the first five research projects mentioned above show that it is clear that lung cancer risk increased with the increasing concentration of arsenic exposure, especially in Chile. In central Italy, the mean concentration of arsenic exposure is 19.3 ug/L, but the hazard ratio (HR) is over 2. Compared with other results under a similar concentration, the risk of lung cancer is higher. Exploring the difference further, we noticed that the average exposure time is 39.5 years in central Italy, far exceeding the exposure time in the other four studies. Therefore, we believe that not only the dose, but also the duration, affects the occurrence of lung cancer caused by arsenic. Upon inclusion of more people, the longer the observation, the closer to reality the modeling: if accurate concentration detection is difficult, then longer data collection is even more difficult. In the last four studies that considered arsenic exposure to be unrelated to the risk of lung cancer, the exposure time was about 10 years, which is far from enough compared to other studies lasting nearly 40 years. Therefore, the analysis of arsenic exposure and the risk of lung cancer may need a longer follow-up. In summary, we have more reason to believe that low to medium concentrations of arsenic are related to the occurrence of lung cancer. Arsenic exposure time and other bias factors also affected the risk of lung cancer. Further research is necessary to provide a more accurate understanding of the relationship between low- to medium-level arsenic exposure and lung cancer.

### Epidemiology of arsenic and lung non-malignant diseases

Except for lung cancer, arsenic exposure may also play an important role in other non-malignant diseases of the lungs. Large-scale population studies have found that arsenic is closely related to lung function [44]: in the low to medium dose range, its harmful effects are obvious, the higher the arsenic concentration, the lower the forced expiratory volume in 1 s (FEV1) and the forced vital capacity (FVC) [44]. Chronic arsenic exposure caused obstructive lung damage, and the severity of that damage increased with increasing arsenic exposure [6, 45]. In Bengal, compared with the normal skin group, the skin lesions caused by high levels of arsenic in the occurrence of chronic cough and chronic bronchitis were more numerous and more severe [46]. In early childhood, exposure to arsenic can increase the mortality of bronchiectasis [47]. The studies suggested that arsenic exposure may play an important role in non-malignant lung disease, though the reports remain limited: it is necessary to study the relationship between, and dose dependence of, arsenic and non-malignant lung diseases.

### Current research on arsenic and lung cancer

Epidemiological studies differ as to whether low to medium concentrations of arsenic are pathogenic, so many people focus on animals and cells. Merrick et al. found that, the incidences of lung cancer in the 50 ppb and 500 ppb groups of lifetime arsenic exposure in CD1 mice were 51% and 54%, respectively, which were significantly higher than that in the control group (22%) [48]. Wang et al. proved that arsenic can induce human lung epithelial cell malignant transformation [49]. Subsequently, under the exposure to arsenic at concentrations of between 0.5 and 2.5 μM and for times ranging from 13 weeks to 26 weeks, many other researchers also found that lung epithelial cells transformed successfully. Studies have shown that the regulation of cell proliferation, apoptosis, angiogenesis, and metastasis play important roles in malignant transformation [49–58]. In addition, the inhibition of deoxyribonucleic acid (DNA) damage repair, DNA methylation, and oxidative stress are also involved in carcinogenesis [58–63]. As mentioned above, tumorigenesis is a complex process: in animal models, arsenic exposure was found to disrupt immune function, and epithelial barrier function [64, 65]. Genetic analysis after intrauterine exposure of mice found that the level of genes related to lung immunity and mucociliary function changed significantly [66]. These changes may be factors initiating tumorigenesis, but there is still a long way from these changes to the occurrence of lung cancer, and the gaps between the two parts are the key points of carcinogenesis in arsenic exposure. Significant changes were found in cell models. Low concentrations of arsenite can induce cell proliferation, which can promote the cell cycle from G1 to S phase, and upregulate the expression of cyclin D1 through activation of the c-Jun N-terminal kinase (JNK1/c-Jun) pathway in human embryonic lung fibroblast (HELF) cell lines [67]. Similarly, in BEAS-2B, low concentrations of arsenite are involved in the malignant transformation of cells by upregulating cyclin D, which was mediated by the p52-Bcl3 complex [68]. MicroRNA (miRNA) also was found to regulate cell proliferation. Inhibition of miR-222 and miR-301a can decrease the proliferation rates of arsenic-transformed (As-T) cells, in which phosphatase and tensin homolog (PTEN) and interleukin 6 and signal transducer and activator of transcription 3 (IL-6/STAT3) signaling are involved, respectively [50, 53]. In As-T cells, reactive oxygen species (ROS) levels are low and have apoptotic resistance. Increasing ROS by inhibiting catalase can restore the apoptosis ability of arsenic-transformed BEAS-2B [69]. Further research showed that high levels of nuclear factor, erythroid 2 like 2 (Nrf2), upregulated the expression of antioxidant proteins catalase and superoxide dismutase, and anti-apoptotic proteins Bcl-2 and Bcl-xl, which reduced ROS production and enhanced the resistance to apoptosis in arsenic-transformed BEAS-2B cells [53, 70]. In addition, under arsenic exposure, IL-6 can regulate Mcl-1 by STAT3 and mediate the binding of Mcl-1 and Beclin 1 to inhibit apoptosis [71]. In angiogenesis, ROS upregulated by arsenic can upregulate the expression of hypoxia-inducible factor 1 (HIF-1) and vascular endothelial growth factor (VEGF) by activating AKT and mitogen-activated protein kinase (ERK1/2) signaling pathways [72]. Under arsenic exposure, HIF-1α accumulated in a dose- and concentration-dependent manner depending on the degree of protein stability, and affected the unanchored growth of transformed cells by mediating glycolysis [73]. Meanwhile, HIF-2α participated in arsenic-induced human bronchial epithelial (HBE) cell transformation by regulating IL-6 and IL-8 [74], and by regulating Twist1 and Bmi1 in epithelial–mesenchymal transition (EMT). Among them, Bmi1 was thought to be related to the maintenance of stem cells mediated by arsenite [75]; However, some studies found arsenic accumulation induced by inhibiting ubiquitination of HIF-2, which participates in the malignant transformation of arsenic-induced cells by inhibiting P53 protein [76]. Those changes are all related to the metastasis of the tumor. Arsenic inhibits DNA repair by suppressing the expression of related genes and inhibiting the base excision repair (BER) and nucleotide excision repair (NER), which is commonly seen in the combined effect of arsenic and other carcinogens, such as benzo[a]pyrene diol epoxide (BPDE), radon and solar ultraviolet radiation [77–81]. DNA methylation that can control gene expression is involved in the occurrence of lung cancer. In A/J mice, arsenic exposure decreased the expression of Ras association domain family member 1 (RASSF1A) by hyper-methylating its promoter region [82]. Similarly, DNA methylation changes were observed in mice exposed to AS for 90 days by whole-genome DNA methylation and gene expression analysis [83]. Except for DNA methylation, arsenic can induce oxidative stress; accordingly, the related oxidant and enzyme, including glutathione (GSH) and gamma-glutamylcysteine synthetase (gamma-GCS), were changed [84]. Although there are few studies of those mechanisms, there is no doubt that these mechanisms have broadened our thinking and further research is necessary to enable a deeper understanding of the pathogenic effects of arsenic.

### Current research on arsenic and lung non-malignant diseases

Arsenic also plays an important role in non-malignant lung diseases. It can impair ATP-mediated Ca^2+^ signaling mechanisms and wound repair through reducing P2Y and P2X receptor function, destroying the innate immunity of airway epithelial cells at concentrations of 10 ppb or 25 ppb [85]. The destruction of innate immune defenses can increase the systemic transport of inhaled pathogens and small molecules, resulting in the increased possibility of viral and bacterial infection in mice after early exposure to arsenic [86, 87]. Besides, for the offspring of C57BL/6 mice after intrauterine exposure to 100 μg/L arsenic, lung tissue genetic analysis shows significant changes in lung development, immunity, and mucociliary function [66], in which lung inflammation and autophagy are involved in the damage of the epithelial barrier and may increase the risk of infection [86, 87]. The relationship between arsenic and lung non-malignant diseases is close. Arsenic may increase the occurrence of diseases by destroying the innate immune system and affecting lung development. The inflammation and autophagy may mediate the occurrence of cancer, but the mechanism of lung disease caused by arsenic exposure is uncertain, especially in non-tumorous lung diseases. Further exploration of the mechanism is significant. Due to the limited research into non-tumorous lung diseases under exposure to arsenic, we sought articles on other diseases related to arsenic exposure, which may provide clues to the relationship between lung disease and arsenic. Arsenic can induce atherosclerosis by upregulating monocyte chemoattractant protein-1 (MCP-1), and tumor necrosis factor α, IL-6 [88, 89]. Arsenic-induced hypertension can be explained by increasing calcium sensitization and vascular endothelial dysfunction [90]. Chronic arsenic exposure can cause malignant transformation of liver epithelium cells, and preneoplastic lesions, including fibrosis, and cirrhosis can also occur, impairing the repair of DNA damage, hyperproliferation, and DNA methylation, all of which may cause the aforementioned diseases [91–94]. Renal diseases [95] and neurological disorders [96] can also be induced by chronic arsenic exposure. In summary, arsenic is a pollutant that can affect many organs, and the various organs of the body are interconnected, work together, and have similarities, which may be useful for us in our analysis of the relationship between lung diseases and arsenic.

### Arsenic metabolism and lung diseases

**Fig. 1:**
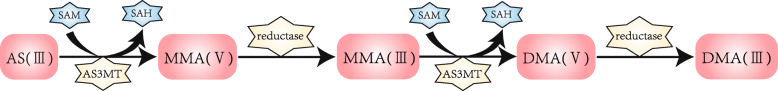
Arsenic metabolism pathway in the human body. 1. iAs (III) is methylated by arsenic-3-methyltransferase (AS3MT), using s-adenosylmethionine (SAM) as a methyl donor to form monomethylarsonic acid (MMAsV). 2. MMAV is reduced to monomethyl arsonous form (MMAsIII), which is then methylated by AS3MT to form dimethylarsinic acid (DMAsV)

To further understand how arsenic is pathogenic, the metabolism of arsenic has to be mentioned. Arsenic exists as inorganic arsenic (iAS) in nature and often accumulates in the human body in various forms after being metabolized. We used to find that the methylation of arsenic is a detoxification process, but recent research indicates that this may be a toxic process [97]. The recognized pathway for arsenic metabolism is that iAs (III) is methylated by arsenic-3-methyltransferase (AS3MT), using s-adenosylmethionine (SAM) as a methyl donor to form monomethylarsonic acid (MMAsV); MMAV is reduced to monomethyl arsenous form (MMAsIII), which is then methylated by AS3MT to form dimethylarsinic acid (DMAsV). DMAsV can be excreted in urine [98] (Fig. 1).

In the female Kunming mouse model, the measurement of the distribution of arsenic in different tissues after a single injection of arsenite found that iAS is the most abundant in the liver and kidney, while the concentration of DMA in the lung and bladder is maximal [99]; therefore, the distribution of iAS and its methylated metabolites may be tissue specific. Similarly, the main metabolite in the lung was found to be DMA [100]. The adult female B6C3F1 mouse model also implied that after a single exposure to arsenate, the percentage of DMA in the lung was the highest, and increased with repeated exposure [101], so DMA may play an essential role in the lungs. Studies have shown that DMA can induce DNA damage, cytotoxicity, chromosomal abnormalities, apoptosis, and gene mutations by inducing oxidative stress [102–104], and its reductive metabolites have been proven to be genotoxic and tumorigenic [105]. In the study of other non-malignant diseases of the lungs, it was found that under the influence of low-dose arsenic exposure, neither iAS nor DMA could change the cytokine secretion induced by *Pseudomonas aeruginosa*. In contrast, MMA increased the secretion of IL-8, IL-6, and chemokine ligand 2 (CXCL2) induced by *Pseudomonas aeruginosa*. These results indicate that MMA may negatively affect the innate immune response of human bronchial epithelial cells to *Pseudomonas aeruginosa* [106]; therefore, we speculate that the two main metabolites of sodium arsenite, MMA and DMA, may play an important role in the development of various diseases in the lungs. To further understand how arsenic works, and what role it plays in the occurrence and development of lung diseases, we need to understand it from a more comprehensive perspective.

## Therapeutic effect

Arsenic, as a traditional Chinese medicine, has a long history of more than 2000 years in China. Arsenic trioxide (ATO), as a chemotherapeutic drug, has been approved for use in relapsed and refractory acute promyelocytic leukemia by the Food and Drug Administration [107]. In addition to leukemia, arsenic is also used in solid tumors, especially in cases of lung cancer [108]; as for other non-malignant lung diseases, arsenic may also play an important role [109–112]. We are going to elucidate those relationships between ATO and lung diseases.

### ATO and lung cancer

**Fig. 2:**
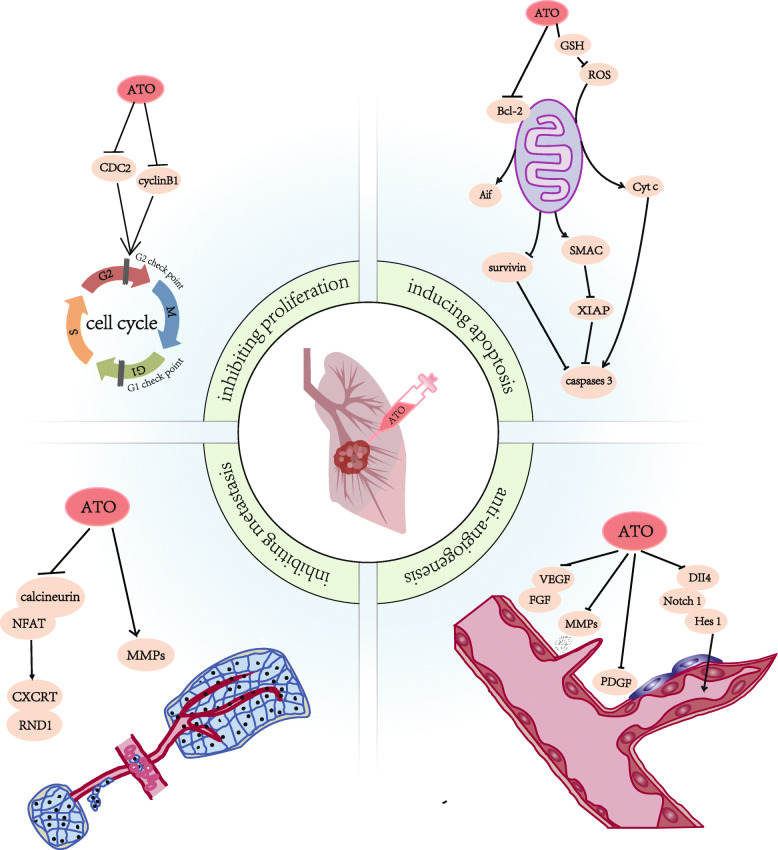
The anti-cancer mechanisms of ATO

In lung cancer, ATO can play an anti-cancer effect by inhibiting cell proliferation [113], inducing apoptosis [114] and anti-angiogenesis [115], and inhibiting tumor metastasis [116], as shown in Fig. 2.

The anticancer mechanisms of ATO involve inhibiting cell proliferation, inducing apoptosis and anti-angiogenesis, and inhibiting tumor metastasis. ATO can inhibit proliferation by downregulating cyclin-dependent protein kinase (CDC2) and cyclinB1, causing cell cycle arrest. ATO treatment can affect mitochondrial function, regulate related molecules, promote the formation of apoptotic bodies, and cause cell apoptosis. ATO can affect CXCRT and RNDI by inhibiting NFAT and calcineurin, thereby inhibiting tumor metastasis. ATO can induce anti-angiogenesis by affecting key molecules of angiogenesis including MMPs and VEGF.

#### ATO and cell proliferation

Cells are an important part of basic experiments, and much research thereon is based on cell experiments. There is no doubt that ATO can inhibit cell proliferation. In the Calu-6 cell model, ATO treatment downregulated CDC2 and cyclinB1, thereby regulating the G2 phase and causing cell cycle arrest [117]. In the SW900 xenograft model, the dose of ATO 7.5 mg/kg can significantly suppress tumor growth compared with the control group [118]. Under the same ATO exposure, the H358 xenograft model confirmed this result; furthermore, the downregulation of thymidylate synthetase (TYMS) protein expression may be associated therewith [119]. Research on the effect of inhibiting cell proliferation in the lungs is limited, but in addition to lung cancer, ATO has been found to inhibit cell proliferation in other diseases. In HepG2 cells, ATO and sorafenib act together to downregulate the expression of cyclin D1, leading to cell arrest at the G0/G1 phase [120]. In Bcr-Abl-positive leukemic cells, ATO and cisplatin can synergistically suppress cell proliferation by inhibiting Myc expression through their influence on ROS generation [121]. In inhibiting proliferative aspects of breast cancer cells, human myelodysplastic syndrome cells [122], and prostate cancer cells [123], ATO can also work alone or in conjunction with other drugs, which may provide new ideas for further studies of the role of arsenic in lung cancer.

#### ATO and apoptosis

In the A549 cell line, ATO treatment revealed apoptosis-related morphological changes including chromatin concentration and formation of apoptotic bodies: the apoptosis-related markers caspase 3 and Bcl-2 were found to change after ATO treatment, and E2F transcription factor 1 (E2F1) may regulate this process [114, 124]. Besides, apoptosis inhibitory gene survival [125] and mitochondrial membrane potential loss [117] were proven to play an important role in ATO-induced apoptosis in the A549 cell model, as well as in the H1355 cell line. ATO was demonstrated to downregulate survivin through the activation of JNK and p38 [126]. In the HI841 cell line, it was further found that GSH and Bcl-2 were downregulated by ATO treatment, which promoted the mitochondrial release of apoptosis-inducing factor (AIF) and SMAC, finally inducing apoptosis [127]. At the same time, ATO also promotes the expression of BACH1 through regulating the level of miR-155, thereby inhibiting the expression of NQO1 and heme oxygenase 1 (HO-1), and inducing cell death [128]. Apoptosis, as an important form of death, plays an important role in the process of tumor treatment and further studies in this way are warranted. How ATO exerts its anti-tumor effect in the apoptosis aspect is of great significance to strengthening its anti-tumor efficacy and reducing drug resistance.

#### ATO and anti-angiogenesis

In terms of anti-angiogenesis, the small cell lung cancer (SCLC) xenograft model shows that ATO can significantly inhibit its angiogenesis, reduce its vascular density, and disrupt the morphological development of its blood vessels. The NCI-H69 cell line model found that ATO treatment can downregulate delta-like canonical Notch ligand 4 (DII4), Notch1, and Hes1, proving that ATO may inhibit notch signaling pathways by targeting Notch1, thus exerting anti-angiogenic effects in SCLC [16]. In addition to affecting the notch signaling pathway, studies in A549 and human umbilical vein endothelial cells (HUVECs) cell lines found that ATO is also involved in other key signals that regulate angiogenesis, including matrix metalloproteinase (MMP)-2, MMP-9, platelet-derived growth factor (PDGF)-BB/PDGF receptor-β, VEGF-A/VEGF receptor 2, and basic fibroblast growth factor (FGF)/FGF receptor-1 [115]. In vivo experiments further confirmed the expression of VEGF-A in ATO transplantable tumor models [129]. Angiogenesis plays an important role in tumorigenesis and development of tumors. Clinically, anti-angiogenic drugs have been proven to exert anti-tumor effects and these have been put into use; therefore, further revealing the mechanism of ATO in inhibiting angiogenesis is of significance to our understanding of the anti-tumor effects of arsenic.

#### ATO and tumor metastasis

HUVECs and human SCLC cell line NCI-H446 have shown that ATO can inhibit the proliferation and migration of endothelial cells. Exploring its mechanism, ATO was found to inhibit the expression of calcineurin, NFAT, and its downstream target genes CXCR7 and Rho family GTPase 1 (RND1), while upregulating the regulator of calcineurin 1 (DSCR1), that is, ATO may inhibit the metastasis of the SCLC by blocking the calcium protein nuclear factor in the activated T cell signaling pathway [116]. Current studies have focused on cell lines; in vivo experiments remain limited, and in short, the evidence of ATO-induced anti-tumor metastasis remains insufficient. Metastasis is a complex process, recent research is incomplete, especially that focusing on arsenic and its role(s) in inhibiting tumor metastasis. Whether arsenic can really play an anti-tumor metastasis function and has clinical significance still needs further research. From laboratory to clinical use, there remains much to be understood.

### ATO and lung non-malignant diseases

In addition to the important role of ATO in lung tumors, ATO also plays an important role in other non-malignant diseases of the lungs. In the pulmonary fibrosis (PF) rat model, ATO inhibits rat PF by upregulating miR-98 and inhibiting its downstream Stat3. Cell experiments further showed that As_2_O_3_ can prevent lung interstitial thickening and inhibit type I collagen and hydroxyproline, thereby preventing the development of PF [109]. In the female BALB/c mouse asthma model, ATO can reduce the severity of asthma attacks. Exploring the mechanism of action thereof may be related to the apoptosis of CD+T cells involved in the ER stress-C/EBP homologous protein pathway [110]. In the model of c immunization, ATO reduces airway responsiveness, airway inflammation, and mucus hyperplasia. Further studies have shown that ATO can cause mitochondrial dysfunction, Ca^2+^-overload, and promote caspase-12 activation, that is, ATO may have important significance in the treatment of asthma [111]. It is also found in the OVA-immunized mouse model that As_2_O_3_ reduces the occurrence of airway hyperresponsiveness (AHR) and cell infiltration into the airway by downregulating the expression of eosinophils. In vitro experiments have further shown that ATO can significantly inhibit the secretion of eosinophil chemokine when it induces a certain apoptosis in primary lung cells [112].

Sodium arsenite and ATO play different roles in lung diseases, but what caused the difference remains unknown. Analysis of cell proliferation, cell cycle distribution, oxidative stress, genetic damage, and apoptotic index of the A549 cell line exposed to sodium arsenite and ATO show that As_2_O_3_ was more cytotoxic than NaAsO_2_. As_2_O_3_ is more effective than NaAsO_2_ in arresting cells in the G2/M phase. As_2_O_3_ is more capable of inducing DNA damage and chromosome breakage than NaAsO_2_ and is more genotoxic. Compared with As_2_O_3_, NaAsO_2_ significantly increased the ROS-level in cells [130]. This result is significant to our understanding of the different roles of arsenic in lung diseases, but further research is necessary to determine how arsenic actually affects lung diseases.

## Conclusions and perspectives

The role of arsenic in the pathogenesis and treatment of the lungs is of great interest, but exactly how arsenic plays a role in the lungs, and why there is such a big difference, is unclear, making further clarification of how arsenic causes and treats the disease may be useful for understanding the occurrence of lung diseases. We found that there are still many unclear aspects of our knowledge concerning the role of arsenic in lung diseases: further research is thus needed. Some researchers believe that arsenic does not directly play a role in pathogenic diseases but enhances the carcinogenic effects of other carcinogenic factors [131]. Arsenic may also work synergistically with other pollutants, causing cancer [132]. Arsenite and benzo[α] can work together to alter metabolism and upregulate glycolysis and oxidative phosphorylation [133]. To elucidate the mechanism of arsenic pathogenicity, animal or cell experiments are often required, but studies have shown that it is difficult to induce tumors after exposure to iAs in rodents, so it may be necessary to explore other, more suitable animal models [134].

ATO treatment is mainly focused on leukemia, and studies on solid tumors are limited. Published research shows that ATO can induce apoptosis of hepatocellular carcinoma (HCC) cells through the generation of ROS [135]. In esophageal carcinoma cells, ATO can induce apoptosis by disrupting the morphology and function thereof [136]. In ovarian carcinoma cells, ATO may induce apoptosis by inhibiting topoisomerase II [137]. All the research reviewed provides novel directions for subsequent studies of the relationship between lung and ATO, but these remain in the laboratory stage, especially with respect to those involving non-malignant lung diseases. Some studies have shown that ATO may increase the sensitivity of radiochemotherapy [138, 139]. Meanwhile, ATO can also increase the therapeutic effects of other drugs, including cisplatin, sulindac sulfide, and the like. The combined effects of ATO and other drugs can influence the cell cycle, apoptosis, and inhibit metastasis [108, 138, 140–142]. Although ATO has been proven to exert anti-cancer effects alone or in combination with other drugs, the specific mechanism of action thereof remains unclear, making it necessary to continue studying the therapeutic effect and mechanism of ATO on lung diseases. In short, further exploration of the relationship between arsenic and the lungs is of great significance for understanding the occurrence of lung diseases and the treatment of lung diseases.

## Data Availability

Not applicable.

## Abbreviations

AHR: Airway hyperresponsiveness
AIF: Apoptosis-inducing factor
APL: Acute promyelocytic leukemia
AS3MT: Arsenic-3-methyltransferase
ATO: Arsenic trioxide
BER: Base excision repair
CDC2: Cyclin-dependent protein kinase
CI: Confidence interval
DMA: Dimethylarsinic acid
DSCR1: Regulator of calcineurin 1
CXCL2: Chemokine ligand 2
FEV1: Forced expiratory volume in 1 s
FGF: Fibroblast growth factor
FVC: Forced vital capacity
HBE: Human bronchial epithelial
HELF: Human embryonic lung fibroblast
HIF-1: Hypoxia-inducible factor 1
HO-1: Heme oxygenase 1
HR: Hazard ratio
HUVECs: Human umbilical vein endothelial cells
IL: Interleukin
MCP-1: Monocyte chemoattractant protein-1
MMA: Monomethylarsonic acid
MMP: Matrix metalloproteinase
NER: Nucleotide excision repair
OR: Odds ratio
OVA: Ovalbumin
PDGF: Platelet-derived growth factor
PF: Pulmonary fibrosis
PPY: Per person-year
PTEN: Phosphatase and tensin homolog
RASSF1A: Ras association domain family member 1
RND1: Rho family GTPase 1
ROS: Reactive oxygen species
RR: Relative risk
SAM: s-Adenosylmethionine
SCLC: Small cell lung cancer
SMR: Standardized mortality ratio
STAT3: Signal transducer and activator of transcription 3
VEGF: Vascular endothelial growth factor
WHO: World Health Organization
